# Acute effects on the psychological perception of university students after participation in the “RegulACTION” workshop for the improvement of emotional health and promotion of an active lifestyle

**DOI:** 10.3389/fpsyg.2024.1419981

**Published:** 2024-06-10

**Authors:** Noelia Belando-Pedreño, Daniel Mendoza-Castejón, Carlos E. López

**Affiliations:** Sport Science Faculty, European University of Madrid, Villaviciosa de Odón, Spain

**Keywords:** emotional regulation, motivation, biopsychosocial model, active lifestyle, interpersonal skills, youth population

## Abstract

**Introduction:**

Prospective research in Health Sciences and Sports Sciences warns of the need to design and implement educational program at the different stages of human development, that promote emotional competences, interpersonal competences, an adequate level of healthy physical activity as well as adherence to the Mediterranean diet and a more active lifestyle on a physical and social level. The main objective of the study was to design an intervention program on intra-and interpersonal competences together with emotional education, nutritional education and healthy physical activity, called ‘RegulACTION’.

**Methods:**

The preliminary study sample consisted of 11 participants aged 21–28 years (*M* = 5.00, *SD* = 8.76) (8 female and 3 males) university students. An *ad hoc* questionnaire was elaborated to evaluate the participant’s perception of the usefulness of the ‘RegulACTION’ experience and a semi-structured interview of 5 questions to assess their perception, identification of their emotions, awareness of their cognitions, emotions and behaviors in the different areas of their lives.

**Results:**

The descriptive results show that the participants are receptive to continuing training in the regulation of cognitions (thoughts) and emotions to increase their satisfaction in different areas of their lives. Regarding the qualitative results, the participants’ perception, collected verbatim, is that they feel the need to apply the knowledge about personal and social emotions, as well as healthy behavior in terms of nutrition and exercise, in their daily lives.

**Discussion:**

The ‘RegulACTION’ program is designed based on the assessment of the prevalence of mental illness in the young adult and adult population, in line with the literature review in the area of knowledge of the theory of emotions, motivational theories and on the occasion of the development of a workshop organized.

## 1 Introduction

Promoting healthy habits is an essential aspect of modern education, especially in the stages of basic training. This concept is progressively integrated into curricula at all educational levels. As per the World Health Organization (2013) the definition of *health*, is a state of complete physical, mental, and social well-being, rather than just the absence of illness. Mental health can be influenced by a variety of complex factors. In order to address this issue, it is necessary to implement comprehensive strategies that involve all social agents. Programs that combine physical activity, emotional work, and motivation development can be helpful in modifying harmful behaviors and increasing satisfaction in acquiring healthy habits. At the age of majority, individuals are often expected to act independently, without external guidance or mentoring. This can sometimes make it challenging for them to receive the support they may need.

The study clearly demonstrates the acute response to the level of importance, commitment, cognitive and emotional perception, and physical exercise techniques implemented in the ‘RegulACTION’ workshop for university students, building on the initial idea.

### 1.1 Theoretical background

The program ‘RegulACTION’ is based on well-established theories that explain human behavior through individual motivation in learning. It enhances the development of daily skills such as ‘knowing how to be’ and ‘knowing how to do’ (MacCann et al., 2020). The Theory of Emotional Intelligence (TEI) (Goleman, 1995), The Self-Determination Theory (SDT) (Deci and Ryan, 2000), the Theory of Planned Behavior (TPB) (Ajzen, 1991) and the biopsychosocial model (Kelly et al., 2009). According to Goleman (1995), emotional intelligence is based on five pillars that explain a person’s emotional behavior: (1) Recognizing one’s own emotions (being aware of one’s own emotions and being able to identify a feeling as it occurs). (2) Emotion management concept (ability to manage one’s feelings so that they are expressed appropriately is based on awareness of one’s emotions). (3) Level of motivation. It is based on the idea that an emotion tends to drive towards an action. Therefore, emotion and motivation are intimately interrelated. Directing emotions, and the consequent motivation, toward the achievement of goals is essential for paying attention, self-motivation, self-management and creative activities. (4) Acknowledging the emotions of others without causing harm or damage to one’s own state of mind or the emotional state of others. In this sense, empathy is a basic psychological construct for identifying, accepting and understanding the emotions of the other person. (5) The ability to relate to others (the ability to interact kindly, positively and effectively with others). In the same line of conceptual analysis of emotional intelligence, Mayer et al. (2000) and Mayer and Salovey (2007) establish four psychological bases analogous to Goleman’s definition, such as: (1) emotional perception (emotions are perceived, identified, valued and expressed); (2) the cognitive dimension of emotion (emotions influence cognition, thoughts); (3) the understanding of one’s own emotions and those of others in order to establish a common understanding of the emotions of others; (4) the understanding of one’s own emotions and those of others in order to establish a common understanding of the emotions of others; (5) emotional management, the understanding of the emotions of others in order to establish a common understanding of the emotions of others. Over the past 3 years, following the pandemic, scientific evidence has focused on demonstrating the importance of emotional regulation in university students as a mediator of mental health in aspects such as body image, depression level (Wu et al., 2023), eating behavior, and coping with social fears (Zhang et al., 2021).

Depending on their level of self-determination (SDT), intrinsic motivation, extrinsic motivation or amotivation, students can increase their engagement in the learning process if their needs for perceived competence, personal autonomy and social relationships are met. In this sense, it has also been shown that the design of learning situations and the motivation of teachers in applying teaching processes directly influence their students’ behavior (Shuo et al., 2022). We can then include the concept of the teacher as a model, where one’s own attitude in the application of programs, such as the use of active and instructional methods based on a guided search with group tasks, could increase the impact on the psychological needs of individuals, as occurs in this program (Aloufi et al., 2021).

When considering the intention to perform an action, an individual’s behavior is influenced by factors such as their predisposition toward carrying out the action and their perception of control over it. This includes their level of expertise and voluntariness in the decision to participate. Furthermore, social factors such as contextual pressure and peer observations can influence an individual’s behavior prior to performance, whether positively or negatively (González Ruiz and Izquierdo Rus, 2023). Therefore, educational proposals that aim to create a positive climate and instil in students the belief that they can successfully change their habits with control and confidence, play a crucial role in the process, as suggested by the Theory of Planned Behavior (TPB). To achieve this, overcoming challenges related to physical activity is essential. Providing positive, interrogative, and prescriptive feedback can help individuals face future tasks or challenges of any kind.

Another important aspect in learning processes is emotional intelligence and its regulation. During training, it is crucial for individuals to develop multidimensional skills and work on socio-emotional capacities to successfully navigate personal areas of growth, as defined by Goleman (1995) in his conception of emotional intelligence. García Fernández and Giménez-Mas (2010) proposed an explanatory model of emotional intelligence that considers cognitive abilities (executive functions), personality traits, and abilities to process emotional information, among other factors. The authors base their application of EI on the study of internal or endogenous aspects, such as particular traits of the individual that can be innate or acquired through learning or knowledge, and external or exogenous aspects, which are behaviors based on adaptation to the environment.

The proposal presented here addresses elements such as responsibility (the ability to understand the consequences that actions may have in an intuited future), common sense or the ability to learn and unlearn, of an endogenous nature. In addition, situations are presented that promote the ability to adapt to the changing environment, empathy (the predisposition to understand what is happening to others and to accompany them in this process) or the ability to communicate assertively (using friendly language that is positive for us). Themselves and with others, thus making room for the external dimension of EI.

Finally, the biopsychosocial model (MBPS) is included as a reference, which aims to healthy habits and an active lifestyle in the population. As mentioned above, the search for a state of overall wellbeing for the individual involves trying to develop the maintenance of comprehensive health throughout the different stages of life. This multifactorial approach to health needs to be promoted from the earliest ages through to adulthood, with particular attention being paid to the behavioral level of the youngest, referring to psychosocial behaviors such as personal and social responsibility, self-management of emotions, empathy, social connectedness, among others (Wright and Burton, 2008; Hellison, 2011). If adequate levels of daily physical activity are not achieved, the help that strategies to increase levels of physical activity can provide in the same classroom, combined with the stimulation of cognitive skills and using the stimulation of emotional intelligence, could alleviate these deficiencies to some extent, as has been demonstrated in young students (Ibaibarriaga-Toset and Tejero-González, 2023), and be associated with better academic performance. However, it seems that in higher education these expectations are lost by ceding the autonomy of these issues to the students themselves, which represents an enormous potential loss for students of this age (Carballo-Fazanes et al., 2020; Guedes et al., 2021).

More physically and cognitively active behaviors, voluntary participation in this type of proposals, can improve the general health status of these students, as well as provide this population with psychological tools that correspond to a greater satisfaction of NPBs (Manzano-Sánchez and Valero-Valenzuela, 2019; Ismailov and Chiu, 2022), preserving the general well-being of the youngest. As previously mentioned, this type of proposal can also focus on the regular participation in physical activities in interaction with the social environment, the improvement of the general physical condition and the perception of achieving optimal real and perceived motor competence or “motor literacy” (integration of knowledge, procedures, attitudes and emotions related to motor behavior), as has been observed in previous educational stages (Quarmby et al., 2019; Whitehead, 2019). This is why it is so interesting to intervene at this crucial stage, when new changes are occurring in students’ living conditions, which can alter such important aspects as personal emotional relationships with a partner, friends or family, personal care (sleep, rest, nutrition, physical condition, mental health) and, of course, trying to address one of the main current problems, obesity (Koliaki et al., 2023), or changes in the level of stress demand on a daily basis, combining occasions, studies and work, and more, as has been demonstrated after the COVID-19 pandemic (Hirano, 2023).

### 1.2 Aim

The main objective of this study was to evaluate the perception of young university students regarding the importance of working on emotional regulation, awareness of cognitions (thoughts), analysis of eating behavior, and physical exercise behavior following their participation in the ‘RegulACTION’ workshop. It was hypothesized that the ‘RegulACTION’ workshop, based on training strategies for cognition, emotional regulation, analysis of emotions and nutrition, and the practice of exercise techniques, would improve the perception of young university students about the importance of working on the cognitive, emotional, nutritional, and physical dimensions contributing to comprehensive well-being.

## 2 Materials and methods

### 2.1 Research design

The research design corresponds to a preliminary study and methodological development design of an educational program. The study develops a quantitative type of research (Sampieri, 2014), a descriptive subtype or approach (collection, analysis and presentation of data through quantitative measures) and a qualitative one for the collection of participants’ perceptions of their experiences.

### 2.2 Participants

#### 2.2.1 Sample recruitment

**Figure 1:**
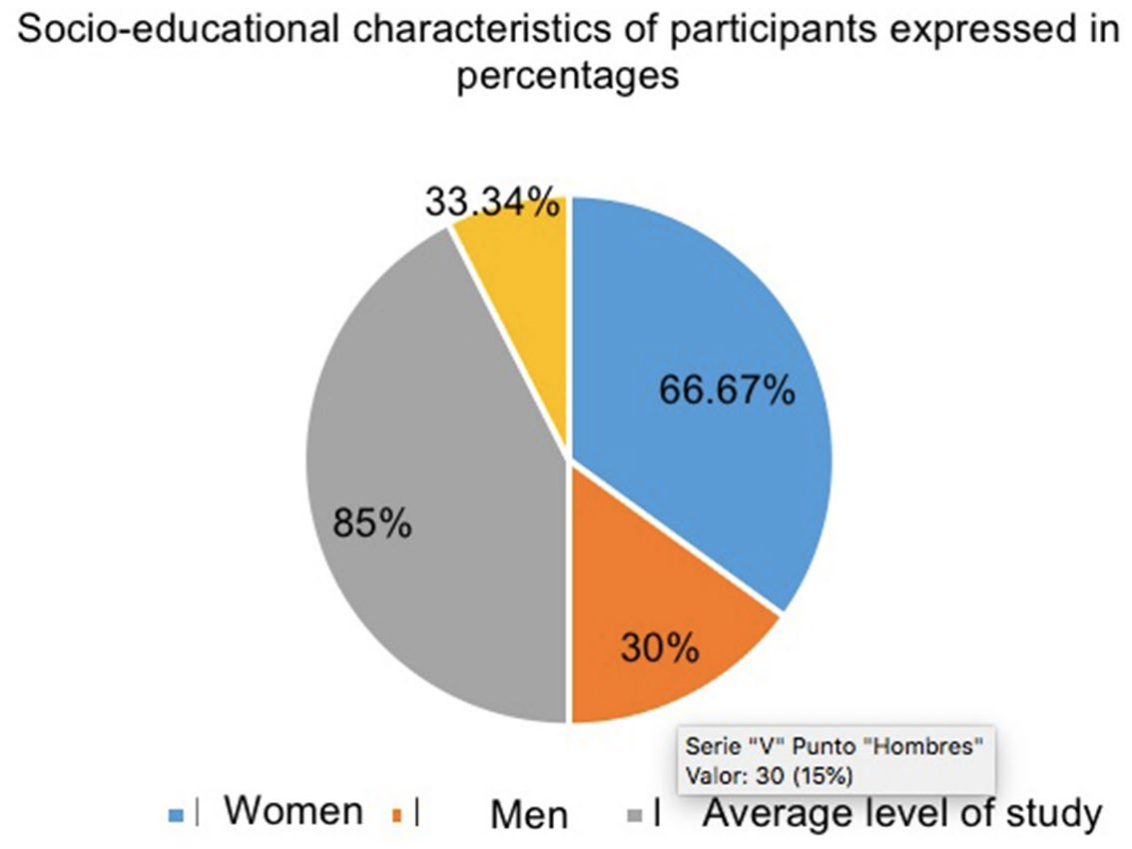
Socio-demographic characteristics of participants instruments.

The sample consisted of 11 participants aged 21–28 years (*M* = 35.00*, SD* = 8.76) (8 female and 3 male) university students from the Community of Madrid (Figure 1). Among the criteria for the selection of participants, the variables of age, gender, educational stage and socio-economic level were taken into account in order to guarantee the representativeness of the population under study. The sampling technique applied was non-probabilistic by accessibility (Otzen and Manterola, 2017). This study has been designed and carried out taking into account all the bioethical principles established by the Belmont Report (1979) and the Declaration of Helsinki (recovered by de Abajo, 2001) principles of autonomy, beneficence, justice and non-maleficence. In addition, this study was approved by the Ethics Committee of the European University of Madrid (code: CIPI/22.299).

#### 2.2.2 Selection criteria

The inclusion criteria (indications given in the workshop registration form), young university students aged 18–35 years without diagnosed mental health pathology and without osteoarticular and muscular resections that prevented them from doing physical exercise.

### 2.3 Variables and measures

An *ad hoc* questionnaire was designed to assess participants’ perceptions of the ‘RegulACTION’ training experience. This instrument combines the collection of quantitative data through 4 items (e.g.: level of importance of the lived experience on emotional regulation and physical exercise in the ‘RegulACTION’ workshop) rated on a Likert-type scale ranging from 1 (not at all or unimportant) to 10 (very important or very important). In addition, it included two open-ended questions, number 5 and number 6, to find out the participants’ perceptions of the transferability of the workshop to different contexts of their lives: What has the experience of the workshop given you in terms of emotional regulation and emotional and physical well-being? What action can you take from now on that will bring you satisfaction with yourself? This questionnaire assesses the participants’ perception of what the “RegulACTION” workshop experience has contributed to them (emotionally, in terms of their relationship with food, and regarding physical exercise behavior). In addition to the reliability test, the content validity of the items was checked by a panel of two experts who assessed whether the content of each item reflected what the researchers wanted to measure. Evidence of validity in relation to the response process was obtained firstly through a cognitive interview in the development phase and secondly from participants’ comments during data collection (Angulo-Brunet et al., 2020).

### 2.4 Procedure

The sample was recruited through registration (online questionnaire in *Google forms* format) on the occasion of the ‘RegulACTION’ workshop, organized as a science outreach activity for the celebration of the European Researchers’ Night 2023. The program was co-ordinated by a maximum of two university professors with a PhD in the field of physical activity and sport sciences and with knowledge in the health sciences (emotional education, motor behavior and nutrition education).

### 2.5 Design and application of the educational program

The educational workshop called ‘RegulACTION’ was structured along eight thematic lines (Table 1) related to emotional skills work, awareness of cognitions (thoughts), nutrition education and physical education (movement techniques) in relation to other curricular subjects. The maximum duration of each workshop was 2 h.

**Table 1:** Structure and contents of the RegulACTION workshop.

Structure	Contextualization	Practical content developed
1st part	Conceptualization and categorization of primary and social emotions.	Activity to identify the biological-adaptive, cognitive and socio-cultural nature of emotions.
2nd part	Motivation model for change (phases)	Activity for the identification of the “phase of the change” in which each participant is: “how you perceive yourself” at the moment on an emotional, physical level and in your social relationship with others
	Psychonutrition activities	The first activity, they had to relate the types of food they consumed with academic or personal situations perceived as stressful. The second activity involved associating an emotion with a type of food and analyzing how they felt after eating that food in relation to the emotion.
3rd part	Body awareness exercises (physical self-concept and identification of physical sensations of emotions). Breathing motor pattern regulation exercises (muscular synergy between diaphragm, abdominal muscles and pelvic floor). Body expression techniques: movements of the different body segments with music.	Practice with different exercise techniques to assess the impact on the participant’s physiological and emotional responses, as well as motor competence (motor ability to respond to each exercise) and perception of fitness.
	Physical exercise techniques organized in the form of a general distribution circuit alternating lower and upper limb actions.	Circuit training organization (20 min.): (a) unipodal and bipodal squat (with and without load); (b) shoulder press (with and without load), (c) biceps curl (with and without load), unilateral traction (unilateral rowing) of upper limbs (with load CE 7–8 [12]).

The “RegulACTION” workshop has been designed based on scientific findings regarding the psychological construct of “intrinsic motivation” analyzed and developed by Reeve (2002) (see Chapter 5, page 83), as well as on the internal and external motives that determine a person’s behavior. The “RegulACTION” workshop also draws on the tenets of various theories that explain human behavior: Selft-Determination Theory (SDT), The Theory of Emotional Intelligence (TEI), Theory of Planned Behavior (TPB) as argued in the Introduction the present study.

Based on the indicated theories as the conceptual foundation, the workshop was structured into 3 parts (view Table 1):

#### 2.5.1 First part

Concepts of emotional intelligence were worked on to identify cognitions (thoughts) and emotions linked to emotional well-being.

#### 2.5.2 Second part

It focused on analyzing motivation for behavior change related to emotions, physical exercise behavior, and eating behavior (psycho-nutritional aspects of eating habits).

#### 2.5.3 Third part

Activities were developed to raise awareness of how emotions are felt in the body, how to care for the body through breathing techniques and exercise techniques. In this part, a strength training session (20 min.) was also conducted, practicing basic exercise techniques.

Applied in activities recognizing different motivational states that students experience in their daily lives, or in activities aimed at understanding the reasons for engaging in sports. Additionally, participants experimented with two psychonutrition activities. In the first activity, they had to relate the types of food they consumed with academic or personal situations perceived as stressful. The second activity involved associating an emotion with a type of food and analyzing how they felt after eating that food in relation to the emotion.

The workshop included physical activities during which participants demonstrated a proactive attitude towards behavior, stimulated by positive, prescriptive, and interrogative feedback (subjective norm) and faced physical challenges that required decision-making within their group of peers (perception of behavioral control). The principles of emotional intelligence (Emotional Intelligence Theory, TEI) were used to work on emotional literacy following Dr. Hitzig’s approach (reflection and discussion on the types of emotions and related attitudes that occur in different situations in the classroom, during play, in sports with friends, at home, in relationships with family, etc.).

### 2.6 Data analysis

A descriptive analysis (means and standard deviations) was carried out on the level of response to items 1 to 4 of the *ad hoc* questionnaire (response range from 1 [not at all/unimportant] to 10 [very important]) and the analysis of reliability (Cronbach’s α and McDonald’s ω, Ventura-León and Caycho-Rodríguez, 2017). To analyze the qualitative data extracted from the *ad hoc* questionnaire. First, the database was cleaned and the Mahalanobis distance was calculated to check for outliers. Next, the students’ textual responses to qualitative items 5 and 6 of the *ad hoc* questionnaires were collected, regarding their perceptions of the importance, commitment, satisfaction and usefulness of the ‘RegulACTION’ workshop. All the information collected was incorporated into a single file stored in Excel 2010. The reliability and descriptive analyses were carried out using Jamovi [The Jamovi Project, (2023). Version 2.4 [computer software]. Obtained from https://www.jamovi.org].

## 3 Results

### 3.1 Quantitative results

Table 2 shows the descriptive results (means, standard deviations and reliability) obtained in items 1 to 4 of the questionnaire to evaluate the experience, the degree of application and the usefulness of the contents worked on in the workshop in other areas of their life (e.g., in physical exercise behavior, in the academic field), using a Likert-type scale of 0–10. The *ad hoc* instrument designed has robust internal consistency values with *α* of 0.82 and *ω* of 0.85 (Ventura-León and Caycho-Rodríguez, 2017). With regard to the most highly valued items, 1 (level of importance of the emotional experience and physical exercise experienced) and 4 (how much they value continuing this type of workshop), with a score of 9.2 out of 10, we found that participants are very receptive to continuing training in managing cognitions (thoughts) and experiencing emotions to increase their satisfaction in different areas of their lives. On the other hand, the score of 9.00 out of 10 in item 2, which deals with the degree of commitment they would acquire to “get going” in order to improve their satisfaction with themselves, is remarkable. Regarding item 3, which also received a high score, the awareness of the flow of thoughts and the relationship with the emotional experience could be more complex.

**Table 2:** Descriptive data and reliability on the assessment of experience in simulated practice.

Items	*M*	*SD*	Cronbach’s alpha	McDonald’s ω
1. Level of importance of the lived experience on emotional regulation and physical exercise from the ‘RegulACTION’ workshop	9.5	0.30	0.82	0.85
2. Degree of commitment to undertake an action that contributes to improving your level of satisfaction with yourself.	9.00	1.02		
3. Level of perception of your thoughts, emotions and way of facing complex daily situations that you have experienced in the ‘RegulACTION’ workshop.	8.00	0.97		
4. How much do you value continuing to do workshops on emotional regulation and active lifestyle on an emotional and physical level?	9.2	1.12		

M, media; SD, standard deviation.

### 3.2 Qualitative results

Table 3 shows the perception of the participants, expressed literally, in response to the following semi-open questions from the *ad hoc* questionnaire: (5) *What has the workshop experience given you in terms of emotional regulation and emotional and physical well-being*? (6) *What action can you take from today that will give you satisfaction with yourself?*

**Table 3:** The following are some of the most representative responses concerning items 5 and 6.

Item	Responses
5. What has the experience of the workshop brought you in relation to emotional regulation and emotional and physical wellbeing?	*Understand the power of emotions* *New ways of avoiding intrusive thoughts.*
	*Be aware of the many internal tools I have to help me improve my emotional life and therefore my physical condition and that of everyone around me.*
	*It is important to understand how to manage intense emotions and recover from them.*
	*Adopting a positive mindset towards oneself and society can be helpful.*
	*I have realized that I need to delve deeper into what I feel and how this affects my daily life. Yes, emotions do influence the amount of exercise I do.*
	*I did not realize that emotions could affect me physically. The workshop has helped me identify it.*
	*The workshop has made me aware that my emotions affect my social relationships.*
6. What action can you do from today that will bring you satisfaction with yourself? Body opening.	Avoid being overly self-critical.
	Refrain from mentally beating oneself up and thinking ‘I am not good enough for what I propose.’
	Develop habits that promote both physical and mental well-being.
	Schedule an activity that involves close companions to share exercise time.
	*I need help from an exercise professional to work on strength and feel better.*
	*I am going to dedicate 2 to 3 times a week to do physical exercise. The workshop has helped me see the need to move and improve my physical condition.*
	*I am going to include more protein in my diet and train for strength at least twice a week with an exercise professional.*

## 4 Discussion

This study aimed to test the importance, level of commitment and perceptions of cognitive and emotional simulation and physical exercise in these university students participating in the ‘RegulACTION’ workshop as an integrative training strategy for adequate emotional education, cognitive perception and promotion of an active lifestyle.

Research has shown that limiting and restrictive thoughts towards oneself can lead to a loss of confidence in the learning process. This has been particularly evident during the COVID-19 pandemic (Romero-Blanco et al., 2020; Gavín Chocano and García Martínez, 2023). This can be very disabling in their academic progress, but by applying rationalization when these thoughts appear, it can be addressed in a better way through critical thinking (Cangalaya Sevillano, 2020). Emotions are a key factor in learning, and negative emotional states can negatively affect the learning process. It is widely acknowledged that emotion and cognition are closely linked. Emotions can have a significant impact on attention, memory, motivation, and decision-making processes. This is particularly relevant in academic research and evaluative testing (Urquijo et al., 2014; Elizondo Moreno et al., 2018). As item 3 suggests, it is crucial for students to learn how to recognize and manage negative thoughts, regulate emotions in personal and social contexts, in order to enhance their well-being and academic performance (Hendrie Kupczyszyn and Bastacini, 2020). The management and regulation of one’s own emotions can contribute to improving perceived self-efficacy (Torre and Lieberman, 2018), reducing uncertainty, and facilitating concentration on critical learning points (Andrés et al., 2017; García-Ros et al., 2023). Regarding the importance and level of commitment of the participants towards using the proposed tools and continuing to explore this type of training (items 4 and 5), they were highly valued. Improving the classroom climate and providing intrapersonal tools for daily life, both inside and outside the classroom, can benefit students in their personal relationships with teachers, classmates, friends, and family. These benefits may also be reflected in their academic performance (Kritikou and Giovazolias, 2022). After revisiting these aspects in the qualitative open questions, the participants expressed that in order to relate adequately to themselves and their environment, they need to become aware of the emotions, the thoughts that lead them to certain emotions that determine their well-being (Jiménez-Parra et al., 2022a,b).

In young university students, it has been shown that there are aspects that can lead to a reduction in the commitment to daily and planned physical activity (restricted schedules, lack of time and lack of will, among others), which have a greater impact on the female sex. Therefore, programs should also focus on this core population (Kortabarria and Galarraga, 2021). With regard to increasing the level of physical activity at these ages (moderate to vigorous intensity), in accordance with the recommended dose (Franklin, 2021), it could contribute to improving psychological aspects such as self-esteem (Aloufi et al., 2021), the level of intrinsic motivation (Rodríguez-González et al., 2021), the development of emotional intelligence (Cazalla-Luna and Molero, 2014) and even better academic performance (Nuevo et al., 2021). Therefore, it would be necessary to continue to provide students with behavioral strategies to promote a healthy and sustainable bond over time with the practice of PE. In this task, educational centers (universities), through student service units and university extension activities, should offer training spaces and supervised physical exercise practice. All this, implemented with other strategies, psycho-nutritional education (healthy behavior with the way of eating) and sleep hygiene for a better rest and impact on the level of attention and perception necessary for cognitive and academic performance in the studies they carry out (Aloufi et al., 2021).

### 4.1 Limitations of the study

Limitations of the study include the selection of the sample, which was based on accessibility and not randomized, which affects the external validity of the study. Another aspect to consider is the research design, which is a descriptive, cross-sectional pilot study. One of the significant limitations of the study is the small sample size so that the data can be extrapolated to the young population. However, we believe that the way in which the sample was recruited is a strength in that the researchers did not know the number of participants and typology (sociodemographic characteristics) of the participants until they carried out the workshop. The only criteria for inclusion (enrollment) in the workshop were a young population between 18 and 35 years of age, university students without diagnosed mental health pathology. Quasi-experimental studies with pre-and post-intervention data collection with experimental and control groups are necessary to verify the causal relationships between the variables analyzed. In terms of data collection, other quantitative questionnaires with psychometric properties validated in the population under study (Cronbach’s Alpha or McDonald’s Omega values) should be administered. Future research should take a quasi-experimental and longitudinal approach, with pre-and post-tests. It would also be necessary to use a randomized probabilistic sampling technique. Based on the observed results and other scientific evidence provided, educational workshops such as ‘RegulACTION’ emerge as an alternative teaching technique based on the incorporation of education in emotional skills, decision making for problem solving and education in a more active lifestyle within and outside of academic performance time. In this way, it contributes to the promotion of positive attitudes toward the various challenges faced by university students, supported by cognitive strategies, emotional regulation actions and the development of physical fitness through the practice of regular and supervised physical exercise. Therefore, this proposal simultaneously promotes emotional and prosocial behaviors (McMullen et al., 2016) and an increase in daily physical activity among young people (Merino-Barrero et al., 2019).

Future longitudinal quasi-experimental studies with repeated measures (pre-test and post-test) are needed, with a non-randomized control group (CG) and an experimental group (EG), analyzed using a quantitative methodology (tests and questionnaires) and a qualitative methodology (observational analysis). In addition, it is intended to check other variables such as (a) anthropometry (percentage of body composition and body perimeters), (b) nutritional status variables (food records, adherence to the Mediterranean diet measured with the *Predimed* questionnaire).

## 5 Conclusion and future practical applications of the RegulACTION workshop

As the main results of the preliminary study developed within the framework of the “RegulACTION” workshop application, a high level of commitment and appreciation of the participants was observed in the activities developed on the analysis of cognitions (thinking), awareness of emotions and the need to do physical exercise on a regular basis. The answers to the semi-open questions revealed verbalizations that showed their ability to reflect on the origin of their emotions in the areas of their lives (personal, domestic, work and social), the ability to become aware of their thoughts, emotions and behaviors, and how this influenced their perception of the world around them. (e.g., “I am aware of the many internal tools I have to help me improve my emotional life and therefore my physical condition and that of everyone around me”).

## Data availability statement

The original contributions presented in the study are included in the article/supplementary material, further inquiries can be directed to the corresponding author.

## Ethics statement

The studies involving humans were approved by Ethics Committee of the European University of Madrid (code: CIPI/22.299). The studies were conducted in accordance with the local legislation and institutional requirements. The participants provided their written informed consent to participate in this study.

## Author contributions

NB-P: Writing – review & editing, Writing – original draft, Validation, Supervision, Software, Project administration, Methodology, Investigation, Formal analysis, Data curation, Conceptualization. DM-C: Writing – original draft, Methodology, Investigation, Conceptualization. CL: Writing – review & editing, Supervision, Investigation, Conceptualization.
